# Enhancer RNAs: mechanisms in transcriptional regulation and functions in diseases

**DOI:** 10.1186/s12964-023-01206-0

**Published:** 2023-08-03

**Authors:** Qianhui Li, Xin Liu, Jingtao Wen, Xi Chen, Bumin Xie, Yang Zhao

**Affiliations:** Department of Obstetrics and Gynecology, Department of Gynecologic Oncology Research Office, Guangzhou Key Laboratory of Targeted Therapy for Gynecologic Oncology, Guangdong Provincial Key Laboratory of Major Obstetric Diseases, The Third Affiliated Hospital of Guangzhou Medical University, No.63 Duobao Road, Liwan District, Guangdong Province Guangzhou City, 510150 People’s Republic of China

**Keywords:** Enhancer, eRNAs, Transcription regulation, Diseases

## Abstract

In recent years, increasingly more non-coding RNAs have been detected with the development of high-throughput sequencing technology, including microRNAs (miRNAs), long non-coding RNAs (lncRNAs), circular RNAs (circRNAs), small nucleolar RNAs (snoRNAs), and piwi-interacting RNA (piRNAs). The discovery of enhancer RNAs (eRNAs) in 2010 has further broadened the range of non-coding RNAs revealed. eRNAs are non-coding RNA molecules produced by the transcription of DNA cis-acting elements, enhancer fragments. Recent studies revealed that the transcription of eRNAs may be a biological marker responding to enhancer activity that can participate in the regulation of coding gene transcription. In this review, we discussed the biological characteristics of eRNAs, their functions in transcriptional regulation, the regulation factors of eRNAs production, and the research progress of eRNAs in different diseases.

Video Abstract

**Supplementary Information:**

The online version contains supplementary material available at 10.1186/s12964-023-01206-0.

## Introduction

Cell ability to respond to specific developmental or environmental stimuli requires strict regulation of gene expression, which is controlled by a variety of genomic regulatory elements, including enhancers, promoters, and silencers. Enhancer is a type of non-coding DNA cis-acting element. The 72-bp sequence, isolated from Simian Virus 40 (SV40) in 1981, is the first enhancer to be cloned, with its function tested [1–3]. Later studies found that enhancers accounted for a large proportion of genomes. Notably, the expression of more than 20,000 coding genes in the human genome may be controlled by hundreds of thousands of enhancers [4, 5]. Mutations or epigenetic modifications in the enhancer region may lead to the occurrence and development of many diseases, including malignant tumors [6–12].

Genomic analysis revealed that many enhancers can be combined by RNA polymerase II (RNA Pol II) and actively transcribed to produce non-coding RNAs, namely eRNAs [13, 14]. A number of studies have shown that eRNAs can play a role in the process of transcriptional regulation [15–19], and the transcription of eRNAs may serves as another sign of enhancer activation [20–25]. The discovery of eRNAs opens up a new pathway for enhancer's mechanism of action, and adds another layer of complexity to transcriptional regulation. This review summarized the currently available information on the progress of research on eRNAs and discussed their biological properties and functions.

### Biological role of enhancers and super-enhancers

During the individual development of eukaryotes, enhancers can activate or enhance gene transcription by binding to transcription factors, cofactors, and chromatin complexes to act on promoters. The dynamic interaction between an enhancer and the corresponding promoter determines its tissue- or cell-specific action mode [26–28]. However, the mechanism of this mode of action has not been elucidated. Activated enhancers usually have several characteristics in common: (1) Enhancers are usually found in the open regions of chromatin [29]; (2) More histone H3 lysine 4 monomethylation (H3K4me1) is detected than histone H3 lysine 4 trimethylation (H3K4me3) [30, 31]; (3) Histone acetylation, such as H3 acetylation at lysine 27 (H3K27ac) [32], and the presence of certain histone variants (such as H2AZ [33, 34]); (4) They can be combined with transcription co-activator, such as Mediator [35], p300/CREB-binding protein (CBP) [36–38] and bromodomain-containing protein 4 (BRD4) [39]. Therefore, chromatin immunoprecipitation and sequencing (ChIP-seq) has been used in many studies for the detection of the aforementioned factors or epigenetic modifications to reveal enhancer activities [30, 40, 41]. However, the annotation of epigenomic features has generated a large number of putative enhancers (> 400,000 to ~ 1 million) in humans, which is tenfold higher than that of coding genes [42–44]. This indicates that additional criteria are needed to more accurately annotate functional enhancers in genomes.

Super-enhancers are a class of cis-regulatory elements with strong transcriptional activation properties, which were first proposed by scholar Richard A. Young in 2013 [45]. They are large clusters of constituent enhancers and usually appear near most of the key genes that determine cell identity. The span range of super enhancers region is usually magnitude higher than typical enhancers [45]. Super-enhancers have higher enrichment densities of transcriptional activation-related histone modifications (H3K27ac, H3K4me1, etc.), Mediator complex, BRD4, p300/CBP and transcription factors than typical enhancers [45, 46]. Like typical enhancers, super enhancers can also be transcribed to form eRNAs, ChIP-Seq results suggested that super-enhancer regions were enriched with more RNA transcription signals than typical enhancer regions, which drove higher levels of target gene expression [46]. Combining the above characteristics, super enhancers have more powerful regulatory functions than typical enhancers.

### Discovery and biological characteristics of eRNAs

Using ChIP-seq, two studies in 2010 found that RNA polymerase II could bind to enhancer regions, and the new non-coding transcripts, eRNAs, were detected through RNA-seq technology [13, 14]. Since then, the existence of eRNAs has been confirmed. Research efforts have been focused on the continuing exploration of their functions in gene transcription regulation [15, 19]. Looking back on the previous decades of research, the existence of eRNAs seemed to be traceable. Before the development of high-throughput sequencing technology, researchers had observed enhancer transcription in β-globin genes [47, 48], human growth hormone genes [49], and major histocompatibility complex class II locus control regions (LCR) [50]. However, no in-depth research was done due to the limited technical resources at that time.

**Fig. 1:**
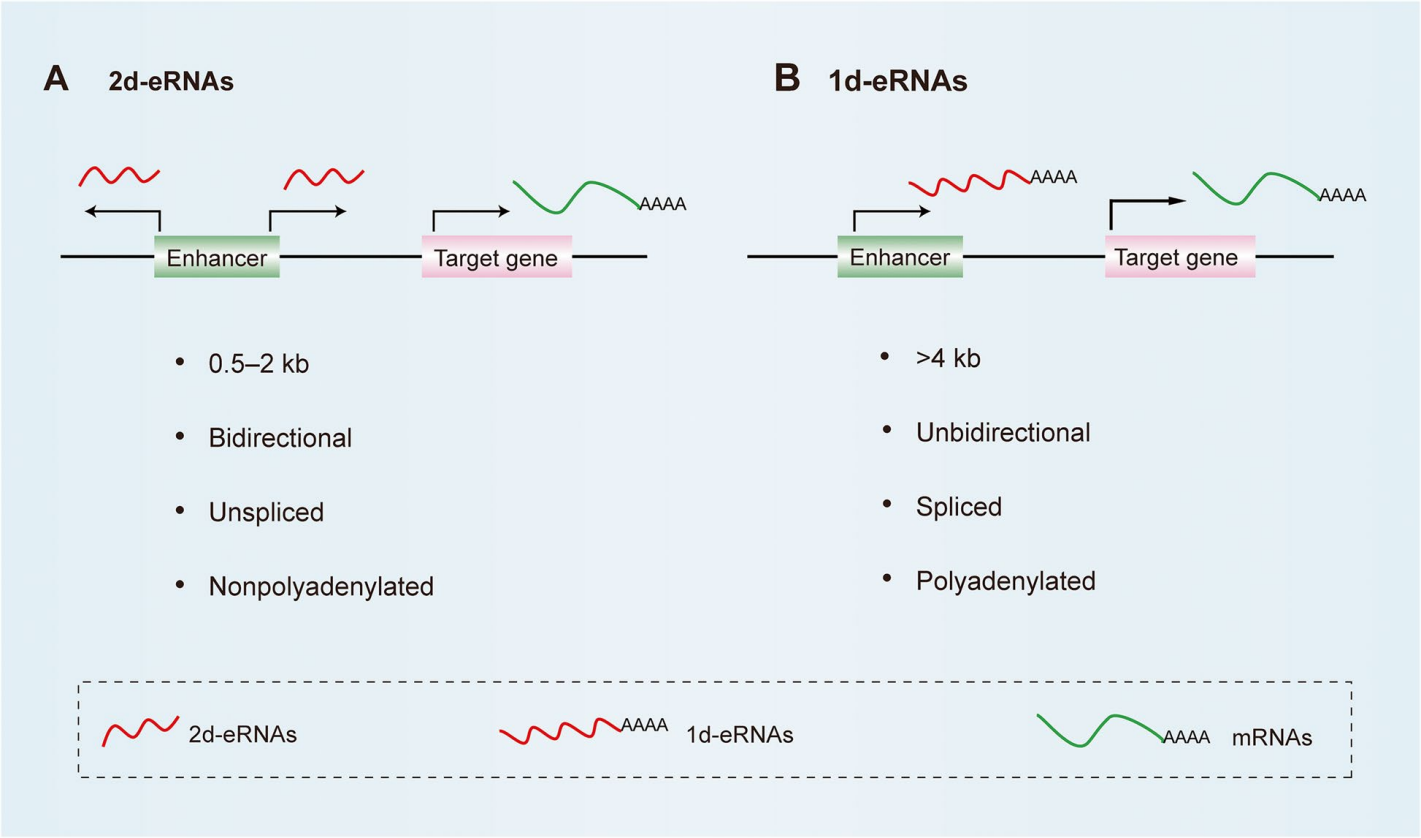
Biological characteristics of eRNAs. **A** Most eRNAs are bidirectional transcribed, which called 2d-eRNAs and with length within 0.5–2 kb, they are unspliced and nonpolyadenylated. **B** About 10% of eRNAs are unidirectionally transcribed (1d-eRNAs), which average length is more than 4 kb, and they are spliced and polyadenylated

According to cap-analysis gene expression (CAGE) technology, the number of eRNAs in humans is approximately 40,000–65,000, which is extremely large [24, 51]. Most eRNAs are bidirectional transcribed, which called 2d-eRNAs, are relatively short in length (within 0.5–2 kb) [52, 53] (Fig. 1A). Such eRNAs do not undergo a complete RNA maturation process. They have a cap structure at the 5′-end but without polyadenylation (polyA) modification at the 3′-end, which also determines their poor stability [13, 19, 53, 54]. Moreover, eRNAs are easily degraded by exosomal complexes in the nucleus [54–56]. Although eRNAs are located predominantly in the nucleus, their abundance is generally low [13, 17, 31]. Certain relatively abundant eRNAs are represented by approximately 0.5–20 copies per cell [57]. In addition, about 10% of eRNAs are unidirectionally transcribed (1d-eRNAs), with an average length of more than 4 kb [24] (Fig. 1B). The structure of this part of eRNAs is comparatively stable, with polyA modification at the 3′-end.

Compared with messenger RNA(mRNAs) and lncRNAs, eRNAs mainly present following characteristics: (a) eRNAs are mainly produced by genomic regions marked by high H3K4me1 and low H3K4me3 histone modifications, whereas the promoter regions of lncRNAs genes usually have high levels of H3K4me3 histone modifications [32]; (b) eRNAs are generally unspliced because their corresponding enhancer sequence does not have a U1 splice site, while both mRNAs and lncRNAs can be spliced [24]; (c) According to research, eRNAs have 90–100-fold lower stability than mRNAs and lncRNAs [13]; (d) The expression of eRNAs is positively correlated with the modification level of the active enhancer marker H3K27ac [31, 58]; (e) The enhancer region producing eRNAs is mainly enriched with the serine-5-phosphorylated RNA Pol II, whereas the promoter region that produces mRNAs binds mainly to serine-2-phosphorylated RNA Pol II [52]. However, studies have found that unidirectional eRNAs (1d-eRNAs) and lncRNAs have similar functions. Currently, it is generally believed that no clear boundary exists between them, that is, many annotated 1d-eRNAs may actually be lncRNAs, and vice versa [59, 60].

### Approaches to detect eRNAs

Although RNA-seq technology has a wide range of applications, it has not been employed on a large scale in the detection of eRNAs mainly due to their poor stability. Methods such as global run-on sequencing (GRO-seq) and its derivative, the precision nuclear run-on sequencing (PRO-seq) have been used to detect the presence of eRNAs, that is, to determine the transcription at a certain stage of cell development by detecting the sequence of new transcripts in the cell [19, 57, 61–65]. Since eRNAs have a 5'-cap structure, CAGE, precision run-on of capped RNA and sequencing (PRO-cap), 5′-end-selected global run-on followed by sequencing (5'GRO-seq) can also be used for detection [19, 24, 66]. In the functional study of eRNAs, short hairpin RNA (shRNA), small interfering RNA (siRNA), locked nucleic acid (LNA), antisense oligonucleotides (ASOs) and dCas9-KRAB-sgRNA technologies were initially used to knock down the expression of eRNAs transcripts in the nucleus [19, 57, 67]. However, some scholars questioned whether RNA interference (RNAi) can really obstruct eRNAs expression [68]. Therefore, researchers used a combination of two knockout methods to ensure the knockout efficiency, such as siRNAs + LNAs [19, 57], siRNAs + ASOs [19, 69], ASOs + dCas9-KRAB-sgRNA [70] etc., or designed two or more siRNAs/ shRNAs/ LNAs/ ASOs to avoid the off-target effects [17, 71, 72]. Besides, overexpression plasmids [70] or CRISPR activation (CRISPR/dcas9 SAM) [71] were applied to induce the expression of eRNAs.

### Functions of eRNAs

#### Serving as a sign of enhancer activity

Distal-acting enhancers are key elements in the regulation of time- and cell type-specific gene expression patterns. The genome-wide identification of active enhancers is necessary to understand gene expression and developmental and disease-related processes. Initially, researchers assessed the enhancer activity mainly by analyzing the recruitment of stimuli-induced transcriptional activators, epigenetic modifications of histones, and the transcription levels of nearby genes. Later studies found that enhancer transcription and the production of bidirectional eRNAs were also closely related to the enhancer activity [21, 73]. In addition, regions of the human genome with both enhancer chromatin markers and eRNAs transcriptional activity have a higher verification rate of enhancer activity than those with enhancers annotated only by chromatin modification data [24]. However, whether eRNAs can reflect the enhancer activity independently of other markers has not yet been established. Currently, it is generally believed that they can reflect the enhancer activity more accurately than other markers [22–25, 74]. Moreover, they may also be reliable predictors of cell type-specific enhancer activity [24]. Therefore, the sensitivity of active enhancer annotation can be improved by combining chromatin modification markers and eRNAs transcription.

#### Promoting the transcription of enhancer target genes

Traditionally, enhancers can be combined with transcription factors, which in turn interact with mediator complexes or other transcription co-activators to promote the recruitment of RNA Pol II and chromatin-modifying enzymes to the promoter region and form chromatin loop (enhancer-promoter loop) to foster the transcription of enhancer target genes. The discovery of eRNAs reveals the complex connection between enhancers that produce eRNAs and target genes, as well as the importance of eRNAs in promoting the transcription of adjacent target genes [15]. In this respect, Kim et al. found that the expression level of eRNAs was positively correlated with that of the coding genes in close proximity [14]. Furthermore, Melo et al. established that p53, a type of important tumour-suppressor proteins, induced target gene transcription by producing eRNAs from distant enhancer regions [16]. In this study, the researchers first identified several p53 binding enhancer regions (p53BER), which have a high affinity with p53, required them to exert their activities, and produced eRNAs in a p53-dependent manner. These authors later found that the suppression of p53-induced eRNAs led to changes in the p53-dependent transcription of the coding gene near p53BER, and the eRNAs expression enhanced the transcriptional activity of the adjacent coding genes. On the other hand, Lam et al. evidenced that the nuclear receptor Rev-Erb in mouse macrophages inhibited gene expression by suppressing the production of eRNAs at the enhancers [19]. These studies confirmed that eRNAs contributed to the transcription of enhancer target genes, indicating that they are not only by-products of gene transcription, but can be classified as a family of regulatory RNAs that enhance gene transcription. With research advances in this area, eRNAs have been found to exert their transcriptional regulatory effects mainly via the pathways described below.

#### Promoting enhancer-promoter interaction

**Fig. 2:**
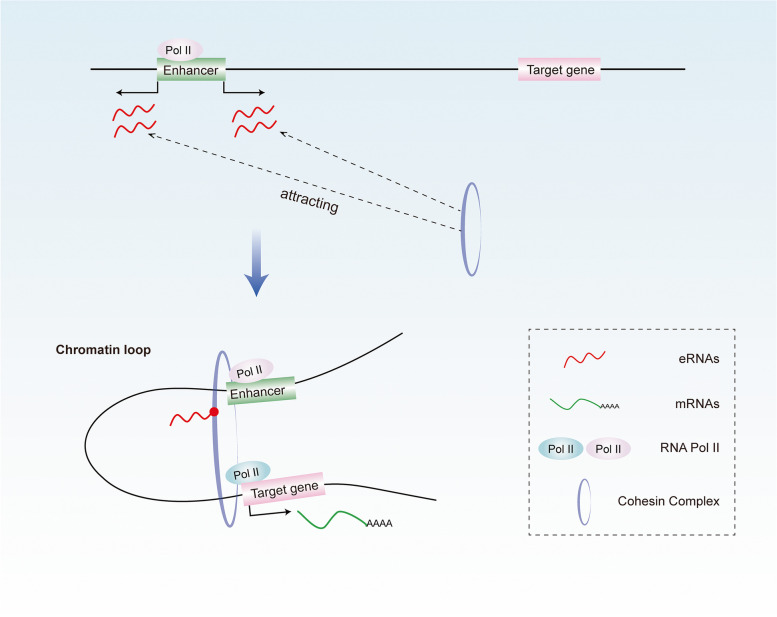
eRNAs can promote enhancer-promoter looping. eRNAs stabilized the enhancer-promoter loops by attracting cohesin complexes, which are key components for the formation and stabilization of chromatin loop structure

Studies have found that eRNAs accelerated the transcription process by fostering enhancer-promoter interaction [57, 67]. For example, Li et al. performed GRO-Seq on cells treated with 17β-estradiol (E2) and observed the correlation between the ER-α binding enhancer, the eRNAs induction, and the upregulated expression of estrogen in the neighboring genes [57]. To investigate whether the eRNAs produced by ER-α binding enhancers affect chromatin structure, they applied a three-dimensional DNA selection and ligation (3D-DSL) program. Interestingly, they found that E2 treatment increased the frequency of enhancer-promoter interactions, while that of targeting eRNAs with siRNA/LNA decreased. In addition, Li et al. also established that eRNAs stabilized the enhancer-promoter loops by attracting cohesin complexes, which are key components for the formation and stabilization of chromatin loop structure [57] (Fig. 2). Consistent with this finding, in another examination, the authors used an anti-Rad21 antibody (a subunit of the cohesin complex) for ChIP-seq. They revealed that a subset of Rad21-binding sites overlapped with the enhancer domain of eRNAs induced by E2, which indicated the presence of an interaction between eRNAs and cohesin complexes. Then, the researchers confirmed their physical combination through RNA immunoprecipitation.

#### Increasing chromatin accessibility

**Fig. 3:**
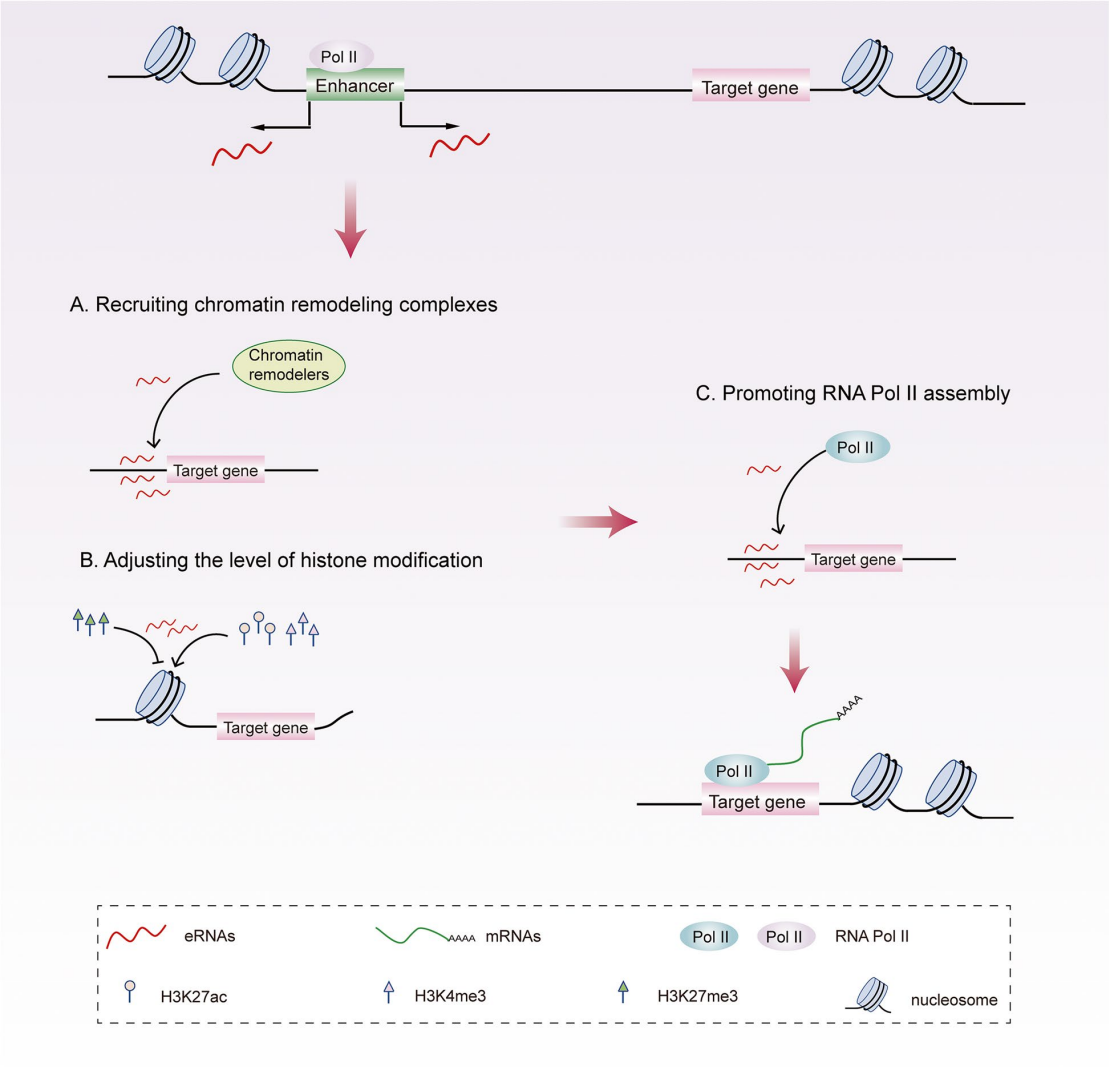
eRNAs can increase chromatin accessibility. eRNAs can form an open chromatin environment by recruiting chromatin remodeling complexes (**A**) or adjusting the level of histone modification in the proximal promoter and enhancer regions (**B**), thus promoting the binding of RNAPII (**C**), then promotes the transcription of target genes

Mousavi et al. found that the myogenic transcription factors MyoD and MyoG combined extensively in extragenic regions [17]. Among them, multiple enhancers upstream of the MyoD1 gene could be transcribed to produce eRNAs, including eRNA produced by a core enhancer (CE) (^CE^RNA) and that by distal enhancers (DRR) (^DRR^RNA). By curbing the expression of eRNAs, the authors found that the reduction in ^CE^RNA levels significantly decreased the occupancy rate of RNA Pol II in the proximal region of the MyoD1 gene. On the other hand, the knockdown of ^DRR^RNA diminished the occupancy rate of the proximal RNA Pol II of the MyoG gene, which is the downstream gene of MyoD1. The authors also assessed the influence of eRNAs on molecular events before RNA Pol II assembly, namely chromatin accessibility. They found that the knockdown of ^DRR^RNA significantly reduced the chromatin accessibility of MyoG. After the decrease in ^CE^RNA expression, the cells were less susceptible to Deoxyribonuclease I (DNase I) treatment at MyoD1 and MyoG. The results of this study shows that eRNAs from the MyoD1 regulatory region promote chromatin recombination/depolymerization and RNA Pol II assembly at specific sites in the muscle-derived gene regulatory network, thereby affecting gene transcription. These findings also suggest that eRNAs can form an open chromatin environment by recruiting key transcription factors and/or chromatin remodeling complexes (Fig. 3A), thus promoting the binding of RNA Pol II, basic transcription factors, and other pre-priming complexes to DNA (Fig. 3C). The research of Tsai et al. confirmed this conclusion [70]. In addition, Pnueli et al. [67]found that the level of active promoter marker H3K4me3 at the target gene promoter was significantly reduced after the knockdown of eRNAs. The level of active enhancer marker H3K27ac at both the promoter and enhancer decreased, but that of repressive histone H3 Lys 27 trimethylation (H3K27me3) increased. It can be seen that eRNAs may also affect the transcription of target genes by adjusting the level of histone modification in the proximal promoter and enhancer regions so as to change the chromatin state (Fig. 3B).

#### Facilitating RNA Pol II pause-release to enhance transcription extension

**Fig. 4:**
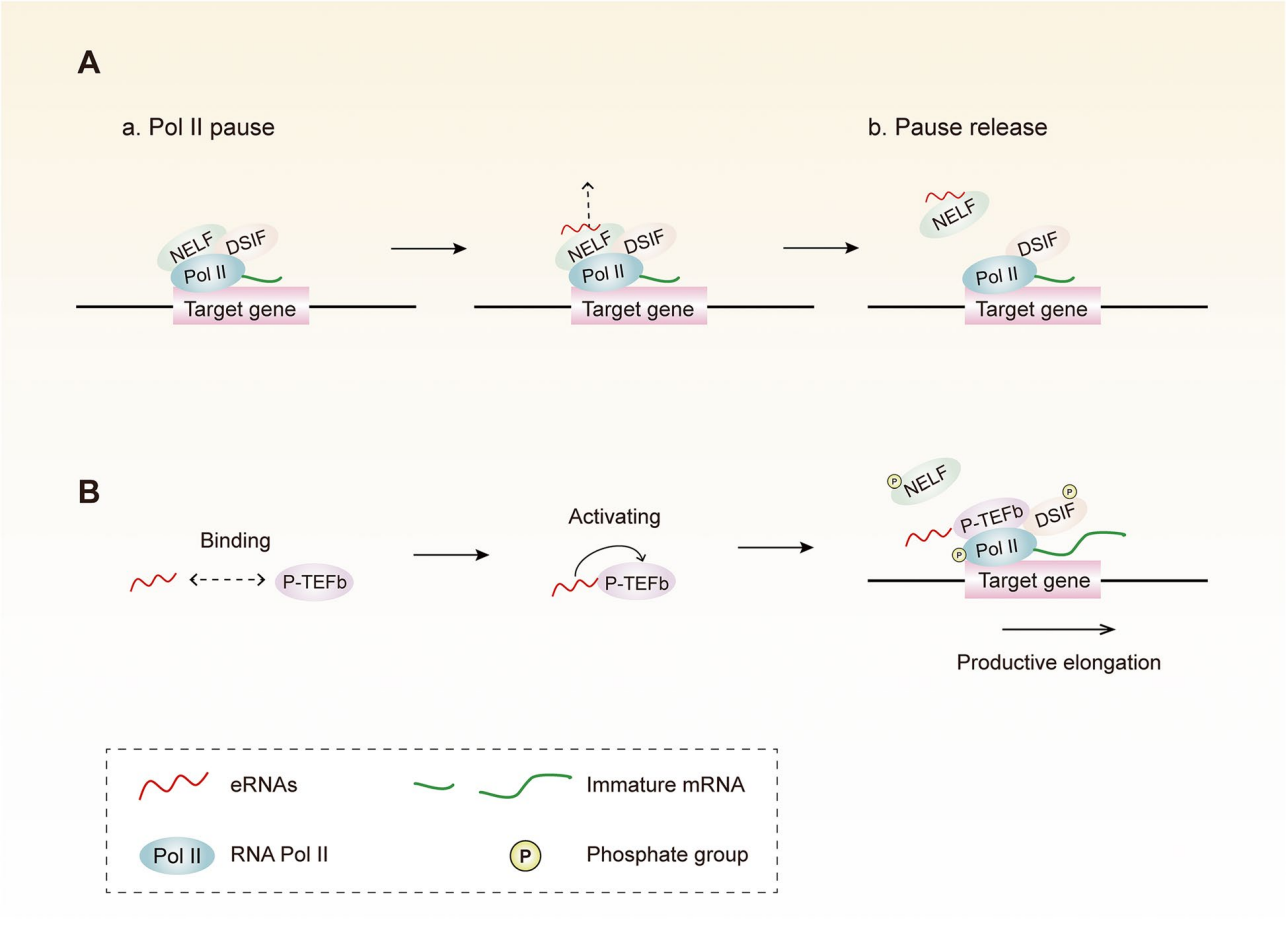
eRNAs can facilitate RNAPII pause-release to enhance transcription extension. **A** eRNAs can promote the release of NELF by directly bind to NELF subunit, allowing the RNAPII to effectively transit to a productive transcription extension. **B** eRNAs can facilitate RNAPII pause-release by binding to P-TEFb complex subunit, activating P-TEFb and promoting RNAPII 2 phosphorylation

RNA Pol II pause is a genome-wide regulatory mechanism of higher eukaryotes with common genes for responses to environmental stimulus [75, 76]. Negative elongation factor (NELF) and DRB sensitivity-inducing factor (DSIF) synergistically trigger RNA Pol II suspension by directly binding to RNA Pol II and nascent RNA [75, 77]. The pause release and subsequent elongation are mediated by the positive transcription elongation factor b (P-TEFb), which phosphorylates the RNA Pol II C-terminal domain (CTD), the DSIF, and the NELF [75, 78]. Schaukowitch et al. [79] found that the suppression of eRNA resulted in decreased expression of specific target genes, whereas the chromosomal loop between the promoter and enhancer was not affected. However, the decline of the eRNA level hindered the effective release of the NELF complex from the promoter of a specific target gene during the transcription induction process, accompanied by a decrease in the extended form of RNA Pol II and target mRNA. Ultraviolet RNA immunoprecipitation (UV-RIP) and in vitro RNA pull-down experiments have shown that the eRNA expressed during neuron stimulation can directly bind to the RNA recognition motif (RRM) of the negative elongation factor complex member E (NELF-E) subunit. Replacing endogenous NELF-E with RRM deletion mutants in neurons significantly reduced the NELF complex binding level at the promoter and mRNA induction. In short, eRNAs can promote the release of NELF by acting as a bait for nascent transcripts, allowing the suspended RNA Pol II to effectively transit to a productive transcription extension (Fig. 4A). Zhao et al. [69] confirmed that Prostate specific antigen (PSA) eRNA activates P-TEFb and promotes RNA Pol II-Ser2 phosphorylation (Pol II-Ser2p) by binding to the subunit CYCLIN T1 of the P-TEFb complex, thereby enhancing the transcription of the target gene, PSA mRNA (Fig. 4B). It becomes evident that eRNAs can affect the target gene transcription by regulating the process of transcription elongation in various ways.

### Regulation of eRNAs production

#### Synthesis

**Fig. 5:**
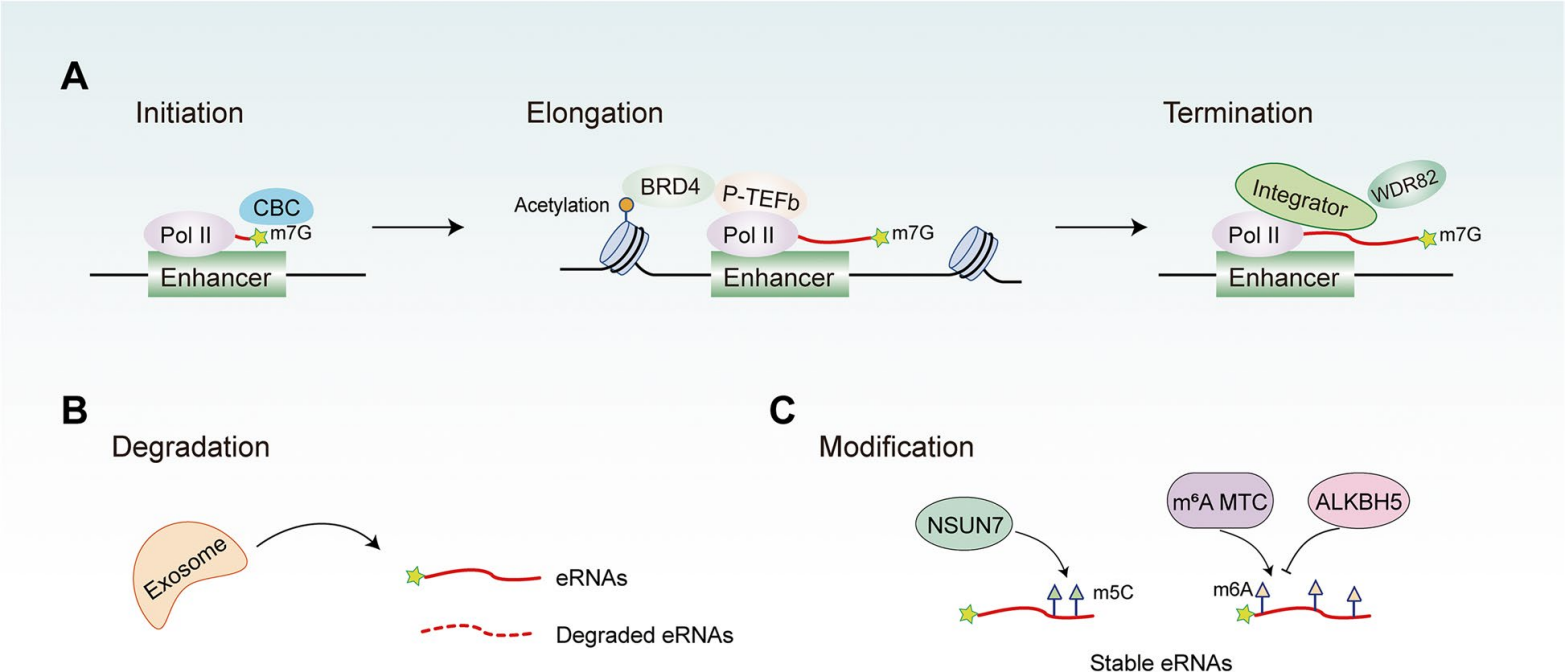
The regulation factors of eRNAs production, degradation and modification. **A** At the initiation of transcription, the cap-binding complex (CBC) form a 7-methylguanosine (m7G) cap at the 5′-end of eRNAs; P-TEFb and BRD4 control the transcript elongation of eRNAs, which are recruited by the acetylated histone tail on the enhancer; The termination of eRNA transcription is mediated by the Integrator complex and WDR82. **B** The degradation of eRNAs is regulated by RNA exosome complexes.** C** Chemical modifications including m^5^C and m^6^A of eRNAs can stabilize eRNAs. eRNAs m^5^C modifications is catalyzed by NSUN7; eRNAs m^6^A methylation and demethylation are catalyzed by MTC and ALKBH5, respectively. MTC: METTL3/METTL14/WTAP m^6^A methyltransferase complex

The production of eRNAs can be regulated by a variety of factors (Fig. 5A). At the initiation of eRNAs transcription, the cap-binding complex (CBC) binds to eRNAs, forming a 7-methylguanosine (m7G) cap at the 5′-end. The transcript elongation of the enhancer is controlled by P-TEFb and BRD4, which are recruited by the acetylated histone tail on the enhancer [78, 80, 81]. The interaction of BRD4 and P-TEFb can release the pausing RNA Pol II, thereby promoting the process of transcript elongation [82]. BRD4 is a member of the bromodomain and extra-terminal domain (BET) family. In the observations of the transcription of eRNAs in the enhancer region centered on the BRD4 peak, Kanno et al. [81] established the small molecule BET inhibitor, JQ1, restrained the elongation of eRNA by facilitating its synthesis, which suggests that BRD4 is involved in the eRNAs transcript elongation process. The termination of eRNA transcription is mediated by the Integrator complex, a multi-subunit complex closely related to the CTD of RNA Pol II that possesses endonuclease activity and necessary for the processing of the 3′-end of the nascent transcript [83]. Lai et al. confirmed that Integrator was recruited to enhancers and super-enhancers in a stimulus-dependent manner [62]. The knockdown of its subunit can reduce the signal-dependent production of eRNAs and eliminate the enhancer-promoter chromatin loop induced by stimulation. Meanwhile, it can also lead to RNA Pol II inability for separation from eRNAs, resulting in the accumulation of RNA Pol II-eRNA complexes, which can decrease the mature eRNAs level. The termination of eRNAs also requires the function of WD repeat-containing protein 82 (WDR82) [84], which is an adaptor protein targeting SET1 H3K4 methyltransferase to chromatin. The knockdown of WDR82 causes defective transcription termination, increasing the abundance and length of eRNAs.

#### Degradation

The degradation of eRNAs is mediated by RNA exosome complexes (Fig. 5B), which consists of nine subunits, six of which form a “loop” structure. The other three subunits containing RNA-binding domains form a “cap” structure. The enzymatic activity of the RNA exosome complex is provided by two additional proteins, Rrp44 (Dis3) and Rrp6 (Exosc10) [85–88]. Pefanis et al. [55] revealed that the expression of non-coding RNAs such as eRNAs increased significantly after the Dis3 and Exosc10 genes were knocked out. During the production of eRNAs, harmful DNA/RNA hybrids, specifically R-loops, may be formed, which may affect the stability of the genome. Among the knockout cells in the Dis3 and Exosc10 genes, Pefanis et al. observed a significant increase in the R-loop structure in the region where eRNAs was transcribed, which suggested that the RNA exosome complex may degrade the harmful secondary structure during the formation of eRNAs to maintain genome stability. Furthermore, another study [24] has detected a 7.4-fold increase in eRNAs peaks after depletion of the exosome complex co-factor MTR4, further confirming that the degradation of eRNAs is mediated by the RNA exosome complex.

#### Modification

Chemical modifications occur on eRNAs can stabilize eRNAs (Fig. 5C), which mainly include 5-methylcytosine (m^5^C) and N6-methyladenosine (m^6^A). Aguilo et al. [89] demonstrated that the NOP2/Sun RNA methytransferase 7 (NSUN7) was a key methyltransferase of eRNAs m^5^C modification. NSUN7 depletion caused an abrupt decrease of eRNAs expression levels and a loss of m^5^C modification on eRNAs. Lee et al. [90] have detected pervasive m^6^A signals on eRNAs by using methylation inscribed nascent transcripts sequencing (MINT-Seq). They identified the m^6^A motif of eRNAs as "GGACT". MINT-Seq results revealed the presence of m^6^A peaks was on the entire eRNA transcript, but with the highest abundance in the middle of the transcript. In addition, m^6^A-marked eRNAs (m^6^A-eRNAs) were longer and possessed higher transcript abundance than non-m^6^A-marked eRNAs. RNA half-life experiments further confirmed that m^6^A-eRNAs were more stable. Mechanistically, m^6^A-eRNAs could recruit the m^6^A reader protein YTHDC1 to active enhancer regions to promote enhancer transcription and facilitate the formation of transcriptional activator condensates, which in turn promote target genes activation. Interestingly, Xu et al. [91] showed that METTL3/METTL14/WTAP m^6^A methyltransferase complex (MTC) and m6A demethylase ALKBH5 located in the enhancer region could regulate eRNAs m^6^A methylation and demethylation, respectively.

### eRNAs in diseases

Studies have gradually revealed the regulatory role of eRNAs in a variety of diseases, such as tumors, inflammation, neurological diseases and muscular diseases. We have summarized the eRNAs with confirmed regulatory role in Table 1.

**Table 1:** eRNAs with confirmed functions in diseases

Diseases	eRNAs	Features	Functions	Ref
Cancer	NET1e	Enrichment of histone H3K4me1 and H3K27ac modification	NET1e contributes to breast cancer progression via upregulation of the important breast cancer oncogene NET1	[71]
	HPSE eRNA	Consisting of three exons, non-polyadenylated	HPSE eRNA promotes cancer progression through driving chromatin looping and regulating hnRNPU/p300/EGR1/HPSE axis	[72]
	KLK3e	Bidirectional, sense (2,170 bp) and antisense (2,230 bp), some of them are polyadenylated	KLK3e facilitates the spatial interaction of the KLK3 enhancer and the KLK2 promoter, enhances long-distance KLK2 transcriptional activation	[92]
	eRNAs produced from p53BERs	~ 600 nucleotides, Nonpolyadenylated	eRNAs produced from p53BERs are required for transcription enhancement of neighboring genes and an efficient p53-dependent cell-cycle arrest	[16]
	PSA eRNA	Enrichment of H3K4me1, H3K4me2 and H3K27ac	The PSA eRNA binds to CYCLIN T1, activates P-TEFb and increases Pol II-Ser2p and cell growth	[69]
	eNEMAL	762 nt, unspliced, polyadenylated	eNEMAL regulates the NEAT1 short/long isoform switch, promotes NEAT1 long isoform expression	[93]
Inflammation	IL1β-eRNA	Unidirectional, with high H3K4me1/H3K4me3	Regulating the LPS-induced inflammatory response in human monocytes	[68]
Neurological diseases	enh3256	-	Regulating GOLPH3L gene expression	[94]
Myogenesis	^CE^RNA, ^DRR^RNA	^DRR^RNA: polyadenylated, single-stranded. ^CE^RNA: bidirectional, highly conserved in mammals	^CE^RNA actives expression of MyoD in *cis*, ^DRR^RNA activates the downstream myogenic genes in *trans*	[17]
	^DRR^eRNA	a ~ 2 Kb transcript, unidirectional, polyadenylated, spliced	^DRR^eRNA directs Cohesin loading in *trans* to regulate Myogenin expression	[70]

*eRNAs* enhancer RNAs, *HPSE* heparinase, *KLK3* Kallikrein-related peptidase 3, *p53BERs* p53-bound enhancer regions, *eNEMAL* eRNA of the NEAT1-MALAT1-Locus, *IL1β* interleukin 1β, *LPS* lipopolysaccharide, *CE* core enhancer, *DRR* distal enhancers

“-” means this part is not mentioned in the article

#### eRNAs in cancer

Enhancers play specific roles in human cells, and their mutations or misregulation can lead to the occurrence of malignant tumors. Therefore, it would be crucially important to understand whether the eRNAs produced by them affect the tumorigenesis process. In a previous study on this topic, Zhang et al. [71] pooled data of large-scale clinical samples and eRNAs expression in tumor cell lines from databases, such as the Cancer Genome Atlas (TCGA), the Cancer Cell Line Encyclopedia (CCLE), Encyclopedia of DNA Elements (ENCODE), Function Annotation of The Mammalian Genome (FANTOM), Roadmap Epigenomics, and 4D Nucleome projects, and found the cancer/lineage-specific expression characteristics of eRNAs. The study identified a total number of 9108 detectable eRNAs in human cancers, which were then divided into three groups: 652 commonly expressed in or above 10 cancer types (ubiquitous eRNAs), and 3124 medium-specific eRNAs expressed in 2–9 cancer types (intermediately specific eRNAs), 5332 eRNAs were expressed in only one cancer type (cancer-type-specific). The ubiquitous eRNAs showed a higher expression level than another two groups.

Meanwhile, a number of studies have confirmed the regulatory roles of eRNAs in tumor progression at the molecular biological level. For example, the HPSE eRNA produced by heparanase can promote cancer progression by driving the chromatin loop and regulating the hnRNPU/p300/EGR1/HPSE axis [72]; Kallikrein 3e (KLK3e), an androgen-induced eRNA, can promote the spatial interaction between the androgen receptor (AR)-dependent gene KLK3 enhancer and KLK2 promoter, and enhance long-distance KLK2 transcription activation [92]. Meanwhile, the study found that KLK3e silencing inhibited the proliferation of prostate cancer cells. Another investigation revealed that eRNAs induced by tumor protein p53 (TP53) are associated with p53-dependent cell-cycle arrest in the breast cancer cell line Michigan Cancer Foundation-7 (MCF-7) [16]. PSA eRNA expression is significantly increased in castration-resistant prostate cancer (CRPC) cells, patient-derived xenografts (PDX), and patient tissues [69]. These findings suggest that eRNAs may be critically involved in tumorigenesis and may thus serve as important biological markers for the diagnosis and treatment of malignant tumors.

#### eRNAs in inflammation

eRNAs also play a role in the progression of inflammation. For instance, Nicholas et al. [68] found that bacterial lipopolysaccharide (LPS) could stimulate human monocytes to produce 76 differentially expressed eRNAs, among which the IL1β-eRNA is associated with the mRNA production of inflammatory mediators IL1β, CXCL8, and IL6. What’s more, the knockdown of IL1β-eRNA decreased the IL1β mRNA expression and protein release induced by LPS, which suggested that eRNAs were important regulators of human innate immune response. Additionally, another study [95] showed that after stimulation of mouse bone marrow-derived macrophages by synthetic glucocorticoids (such as dexamethasone), eRNAs could be generated at the GR binding site (GBS), termed GR eRNAs. The expression of these GR eRNAs showed glucocorticoid-responsive tissues specific features. They also found that after dexamethasone stimulated macrophages, 81 GR eRNAs were up-regulated, and 108 GR eRNAs were down-regulated. The expression of GR eRNAs and its adjacent genes were also significantly correlated, down-regulated genes adjacent to GBSs with eRNA transcription are mostly enriched in biological processes related to inflammatory response processes such as ‘leukocyte migration’.

#### eRNAs in neurological diseases

Yao et al. [96] discovered a set of eRNAs specifically expressed in human brain tissues, and constructed a co-expression interaction network of eRNAs-target genes in the human fetal brain and multiple adult brain regions. They also showed that the active enhancer region with the ability to transcribe brain-specific eRNAs was rich in genetic variants associated with the autism spectrum disorder (ASD). Meanwhile, Hauberg et al. [94] also compared 118 differentially transcribed eRNAs in schizophrenia (SCZ) patient samples with control samples and identified schizophrenia-associated gene/ eRNA co-expression modules from the former group. Another study [97] demonstrated that compared with the sham control group, 77 eRNAs were significantly induced in the stroke group, of which 55 eRNAs were exclusively induced in the stroke group. They randomly selected two eRNAs which were significantly induced in the stroke group for knockdown experiment in vivo. Results showed that infarct volumes in the eRNAs knockdown group were larger than the control group, suggesting that eRNAs were associated with the post-stroke neuroprotective response.

#### eRNAs in myogenesis

As we mentioned above, Mousavi et al. [17]. revealed that ^CE^RNA could active MyoD expression in *cis* by promoting chromatin remodeling and increasing RNA polymerase II occupancy and DNA accessibility, which was a crucial transcription factor in myogenesis. Tsai et al. [70] demonstrated that ^DRR^eRNA which was transcribed from the distal enhancer of MyoD (located on mouse chromosome 7) did not regulate the expression of MyoD, but localized to Myogenin locus (located on mouse chromosome 1) and regulated the expression of Myogenin in *trans*. Furthermore, they found that ^DRR^eRNA could bind to SMC3, which was one member of Cohesin complex, and recruit Cohesin at Myogenin in differentiated C2C12 cells, thus promoting myotube differentiation.

### Perspectives

It is only more than a decade since the discovery of eRNAs. Although increasing research on eRNAs has been performed in recent years and many of their biological functions have been revealed and confirmed, there are still issues that need to be addressed. For example, most of the functions of eRNAs reported in those literature cannot exist independently of enhancers. Thus, it is critical to elucidate whether they can serve as functional genes similarly to other non-coding RNAs, because most eRNAs have poor stability. In addition, it is worthwhile to establish and develop better tools for the manipulation of eRNAs expression, which would be crucial to the success of functional eRNAs research. In addition, although Zhang et al. has suggested the widespread presence of eRNAs in tumor cells through database integration, the specific mechanisms by which eRNAs function in malignant tumors remain to be explored. Furthermore, the lineage-specific expression signatures of eRNAs may guide the molecular typing and diagnosis of tumors. Their transcriptional regulatory role in tumor genes also suggests the potential to be employed as a target for tumor diagnosis or therapy. In conclusion, many unknowns remain regarding the biological functions and specific mechanisms of eRNAs activities, which require further continuous exploration.

## Data Availability

Not applicable.

## Abbreviation

miRNAs: MicroRNAs
lncRNAs: Long non-coding RNAs
circRNAs: Circular RNAs
snoRNAs: Small nucleolar RNAs
piRNAs: Piwi-interacting RNA
eRNAs: Enhancer RNAs
SV40: Simian virus 40
RNA Pol II: RNA polymerase II
H3K4me1: Histone H3 lysine 4 monomethylation
H3K4me3: Histone H3 lysine 4 trimethylation
H3K27ac: Histone H3 acetylation at lysine 27
CBP: CREB-binding protein
BRD4: Bromodomain-containing protein 4
ChIP-seq: Chromatin immunoprecipitation and sequencing
LCR: Locus control regions
CAGE: Cap-analysis gene expression
polyA: Polyadenylation
mRNAs: Messenger RNA
GRO-seq: Global run-on sequencing
PRO-seq: Precision nuclear run-on sequencing
PRO-cap: Precision run-on of capped RNA and sequencing
5'GRO-seq: 5′-End-selected global run-on followed by sequencing
shRNA: Short hairpin RNA
siRNA: Small interfering RNA
LNA: Locked nucleic acid
ASOs: Antisense oligonucleotides
RNAi: RNA interference
p53BER: P53 binding enhancer regions
3D-DSL: Three-dimensional DNA selection and ligation
DNase I: Deoxyribonuclease I
H3K27me3: Histone H3 Lys 27 trimethylation
NELF: Negative elongation factor
DSIF: DRB sensitivity-inducing factor
P-TEFb: Positive transcription elongation factor b
CTD: C-terminal domain
UV-RIP: Ultraviolet RNA immunoprecipitation
RRM: RNA recognition motif
NELF-E: Negative elongation factor complex member E
PSA: Prostate specific antigen
Pol II-Ser2p: RNA Pol II-Ser2 phosphorylation
CBC: Cap-binding complex
m7G: 7-Methylguanosine
BET: Bromodomain and extra-terminal domain
WDR82: WD repeat-containing protein 82
m^5^C: 5-Methylcytosine
m^6^A: N6-methyladenosine
NSUN7: NOP2/Sun RNA methytransferase 7
MINT-Seq: Methylation inscribed nascent transcripts sequencing
MTC: METTL3/METTL14/WTAP m^6^A methyltransferase complex
TCGA: The Cancer Genome Atlas
CCLE: The Cancer Cell Line Encyclopedia
ENCODE: Encyclopedia of DNA Element
FANTOM: Function Annotation of The Mammalian Genome
KLK3e: Kallikrein 3e
AR: Androgen receptor
MCF-7: Michigan Cancer Foundation-7
CRPC: Castration-resistant prostate cancer
PDX: Patient-derived xenografts
LPS: Lipopolysaccharide
GBS: GR binding site
ASD: Autism spectrum disorder
SCZ: Schizophrenia
